# Can preoperative and postoperative CA19-9 levels predict survival and early recurrence in patients with resectable hilar cholangiocarcinoma?

**DOI:** 10.18632/oncotarget.17336

**Published:** 2017-04-21

**Authors:** Jun-Ke Wang, Hai-Jie Hu, Anuj Shrestha, Wen-Jie Ma, Qin Yang, Fei Liu, Nan-Sheng Cheng, Fu-Yu Li

**Affiliations:** ^1^ Department of Biliary Surgery, West China Hospital of Sichuan University, Chengdu, Sichuan Province, China; ^2^ Department of General Surgery, Gandaki Medical College, Pokhara, Nepal

**Keywords:** hilar cholangiocarcinoma, CA19-9, prognosis, early recurrence

## Abstract

**Background:**

To investigate the predictive values of preoperative and postoperative serum CA19-9 levels on survival and other prognostic factors including early recurrence in patients with resectable hilar cholangiocarcinoma.

**Results:**

In univariate analysis, increased preoperative and postoperative CA19-9 levels in the light of different cut-off points (37, 100, 150, 200, 400, 1000 U/ml) were significantly associated with poor survival outcomes, of which the cut-off point of 150 U/ml showed the strongest predictive value (both *P* < 0.001). Preoperative to postoperative increase in CA19-9 level was also correlated with poor survival outcome (*P* < 0.001). In multivariate analysis, preoperative CA19-9 level > 150 U/ml was significantly associated with lymph node metastasis (OR = 3.471, 95% CI 1.216–9.905; *P* = 0.020) and early recurrence (OR = 8.280, 95% CI 2.391–28.674; *P* = 0.001). Meanwhile, postoperative CA19-9 level > 150 U/ml was also correlated with early recurrence (OR = 4.006, 95% CI 1.107–14.459; *P* = 0.034).

**Materials and Methods:**

Ninety-eight patients who had undergone curative surgery for hilar cholangiocarcinoma between 1995 and 2014 in our institution were selected for the study. The correlations of preoperative and postoperative serum CA19-9 levels on the basis of different cut-off points with survival and various tumor factors were retrospectively analyzed with univariate and multivariate methods.

**Conclusions:**

In patients with resectable hilar cholangiocarcinoma, serum CA19-9 predict survival and early recurrence. Patients with increased preoperative and postoperative CA19-9 levels have poor survival outcomes and higher tendency of early recurrence.

## INTRODUCTION

Hilar cholangiocarcinoma (HCCA) is featured by advanced tumor biology, low surgical resectability, high recurrence and poor survival outcome [1–4]. On account of the absence of particular related symptoms in early stage, most patients with HCCA manifest locally advanced disease and unresectable when diagnosed [5]. With the progress of surgical techniques, more and more patients are eligible for curative surgery [6], which furnishes the only possibility of long term survival [7, 8]. However, the prognosis of HCCA is still deplorable [9–11].

Serum carbohydrate antigen 19-9 (CA19-9) has been extensively applied in conventional clinical practices for diagnosis, treatment options, prognosis and therapeutic response detection in HCCA [12–14]. Nevertheless, the prognostic effect of CA19-9 levels on survival remains disputable: several investigators have reported increased preoperative CA19-9 levels predict poor survival outcome [15–17], while others noted that preoperative CA19-9 has no specific association with survival outcome [18, 19]. In addition, the majority of patients with HCCA have hyperbilirubinemia, and hyperbilirubinemia leads to a significant increase in preoperative CA19-9 level [15, 16, 20]. However, most previous studies have ignored the role of hyperbilirubinemia on CA19-9 level, and if patients with hyperbilirubinemia were taken into consideration, the results may be biased. Furthermore, serum CA19-9 concentration is determined by the Lewis phenotype on red blood cells [16, 21]. However, about 5%–14% of the population are Lewis^a–b–^ and these individuals cannot produce CA19-9 (CA19-9 nonsecretory) when they suffer from malignant diseases [4]; even after curative resection, these patients tend to have poor prognosis. Previous studies have also overlooked this significant factor and have not rule out these patients when examining the prognostic effect of CA19-9 levels on HCCA. In view of the above factors, prognostic effect of preoperative CA19-9 levels on survival in previous studies should be modified. Moreover, the prognostic role of postoperative CA19-9 levels and the preoperative to postoperative CA19-9 changes on survival and early recurrence have merely not been investigated in former studies.

The aim of the present study was therefore to retrospectively analyze the prognostic values of preoperative and postoperative CA19-9 levels on survival outcome after curative resection of HCCA in those without CA19-9 nonsecretory and hyperbilirubinemia. Preoperative to postoperative CA19-9 changes on survival were simultaneously analyzed. Furthermore, we also assessed the relationships of preoperative and postoperative CA19-9 levels with various clinicopathological factors including early recurrence (recurrence < 12 months after curative surgery) by using the univariate and multivariate methods.

## RESULTS

### Patient characters

Table 1 outlined the fundamental clinicopathological features of the patients. The median preoperative total bilirubin level after biliary drainage was 23.9 umol/l, and the corresponding median preoperative CA19-9 level was 129.6 U/ml. The median postoperative CA19-9 level was 36.1 U/ml. All patients were operated on with curative surgery with postoperative R0 (87 cases) and R1 (11 cases) margin status. The postoperative complications after curative surgery were detected in 22 patients, which includes bile leakage (8 cases), lung infection (4 cases), hemorrhage (3 cases), peritoneal cavity infection (3 cases), hepatic failure (2 cases), sepsis (1 case), and acute cardiac failure (1 case). No postoperative deaths were detected among the 98 patients, and all of the patients were well followed-up. We examined the early recurrence in 28 of the 98 patients at the first year after surgery, of which local recurrence (*n* = 13) and liver metastasis (*n* = 8) were most commonly to see. The median overall survival time of the 98 patients after curative surgery was 36.8 months with a 1-, 3-, 5-year survival rate of 83%, 51%, and 30% respectively.

**Table 1:** Clinicopathologic characters of 98 hilar cholangiocarcinoma patients treated by curative resection

Variables	*n* = 98
Age, years*	60 [36–79]
Sex (male/female)	51/47
Preoperative TB level, umol/l^*,†^	23.9 [7.1–34.2]
Preoperative ALT level, U/L*	109 [13–720]
Preoperative AST level, U/L*	94 [14–387]
Preoperative Albumin level, g/L*	38.1 [31–50.1]
Preoperative CA19-9 level, U/ml*	129.6 [11.7 – > 1000]
Preoperative CA125 level, U/ml*	20.1 [2.5–120.9]
Preoperative CEA level, ng/ml*	2.7 [0.2–38.2]
Total hospital stay, days*	19 [10–113]
Preoperative biliary drainage (no/yes)	83/15
Portal vein embolization (no/yes)	84/14
Tumor extent (Bismuth-Corlette classification)	
Type I and II	61 (62.2)
Type III and IV	37 (37.8)
Surgical procedures	
Bile duct resection	11 (11.2)
left hemihepatectomy	46 (46.9)
right hemihepatectomy	30 (30.6)
left trisegmentectomy	6 (6.1)
right trisegmentectomy	3 (3.1)
mesohepatetctomy	2 (2.0)
caudate lobectomy (no/yes)	24/74
Portal vein resection and reconstruction (no/yes)	13/85
AJCC 7th T Stage	
T1 and T2	70 (71.4)
T3 and T4	28 (28.6)
Tumor size*	2.9 [1–6]
Postoperative CA19-9 level*	36.1 [7.2 – > 1000]
Tumor resection margin (R0/R1)	87/11
Lymph node metastasis (no/yes)	58/40
Tumor differentiation (well/moderate/poor)	27/46/25
Vascular invasion (no/yes)	82/16
Complications (no/yes)	76/22
Early recurrence (no/yes)^&^	70/28
Survival time, months*	36.8 [5.6–84.8]

^*^ indicated as median and range; ^†^indicated as the bilirubin level just before the surgery; ^&^indicated as recurrence < 12 months after curative surgery; TB: total bilirubin; ALT: alanine aminotransferase. AST: aspartate transaminase; CA19-9: carbohydrate antigen 19-9; CA125: carbohydrate antigen 125; CEA: carcino embryonie antigen; AJCC: American Joint Committee On Cancer.

### Preoperative and postoperative CA19-9 levels on survival

**Figure 1:**
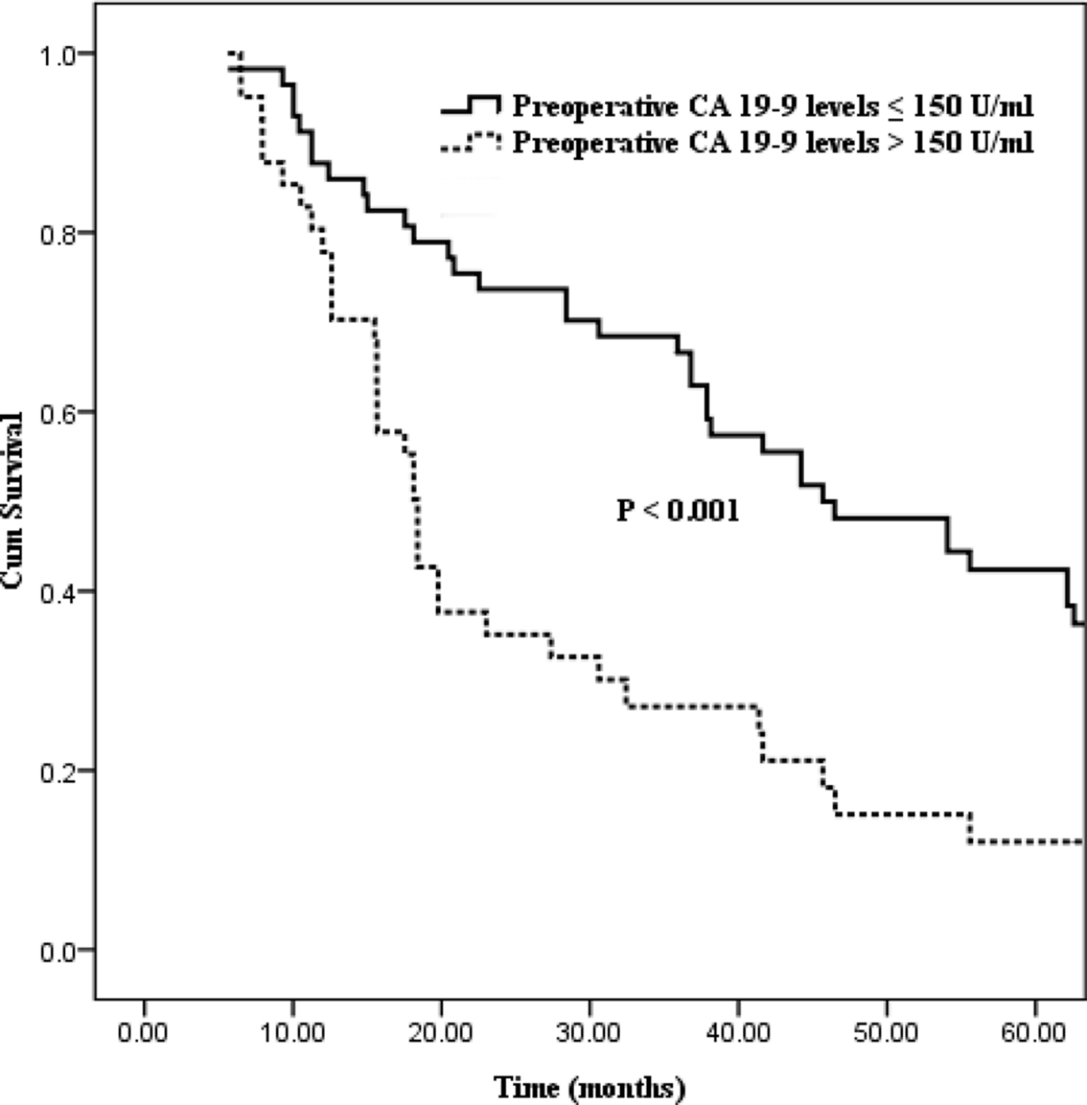
Kaplan–Meier curves comparing survival status stratified by preoperative CA19-9 levels (≤ 150 U/ml vs. > 150 U/ml; *P* < 0.001)

**Figure 2:**
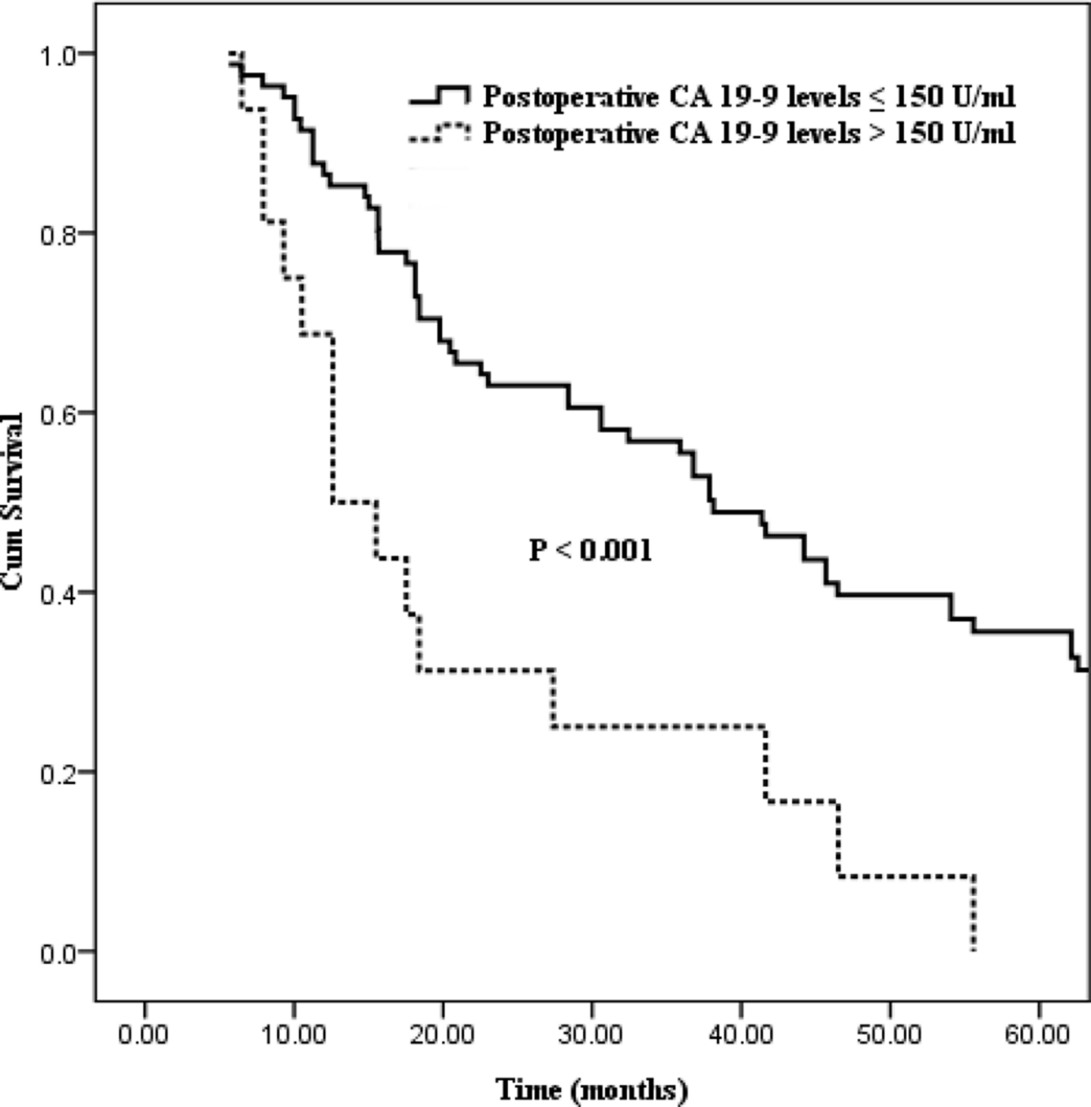
Kaplan–Meier curves comparing survival status stratified by postoperative CA19-9 levels (≤ 150 U/ml vs. > 150 U/ml; *P* < 0.001)

Differences in survival outcome were statistically significant between groups divided in the light of the six preoperative CA19-9 cut-off points (37, 100, 150, 200, 400, 1000 U/ml, the corresponding *P* values of which were 0.035, 0.028, < 0.001, 0.026, 0.010 and 0.010 respectively; Table 2). Excluding the cut-off points of 400 and 1000 U/ml with fewer number of patient cases, the preoperative CA19-9 cut-off point of 150 U/ml showed the strongest predictive value (Figure 1, *P* < 0.001). And then, statistical differences in postoperative CA19-9 levels divided on the basis of the six cut-off points were also confirmed (cut-off points: 37, 100, 150, 200, 400, 1000 U/ml, the corresponding *P* values were 0.004, 0.013, < 0.001, 0.006, < 0.001 and < 0.001 respectively; Table 2). Similarly, the postoperative CA19-9 cut-off point of 150 U/ml was also regarded as the strongest predictive value associated with survival (Figure 2, *P* < 0.001).

**Table 2:** Differences of overall survival in two groups divided on the basis of six CA19-9 cut-off points for 98 patients treated by curative resection for hilar cholangiocarcinoma

	Preoperative CA19-9 levels			Postoperative CA19-9 levels		
CA19-9 cut-off point*	Patients number	Median survival (Months)	*P* value	Patients number	Median survival (Months)	*P* value
≤ 37	10	63.6	0.035	55	44.2	0.004
> 37	88	30.6		43	18.4	
≤ 100	31	54.1	0.028	71	38.1	0.013
> 100	67	28.4		27	17.9	
≤ 150	57	45.7	< 0.001	82	38.1	< 0.001
> 150	41	18.4		16	12.6	
≤ 200	82	37.9	0.026	85	37.6	0.006
> 200	16	15.5		13	15.2	
≤ 400	89	36.8	0.010	92	37.8	< 0.001
> 400	9	10.5		6	7.9	
≤ 1000	94	36.7	0.010	97	36.8	< 0.001
> 1000	4	7.9		1	6.5	

^*^ the unit is U/ml; CA19-9: carbohydrate antigen 19-9.

### Preoperative to postoperative CA19-9 alterations on survival

**Figure 3:**
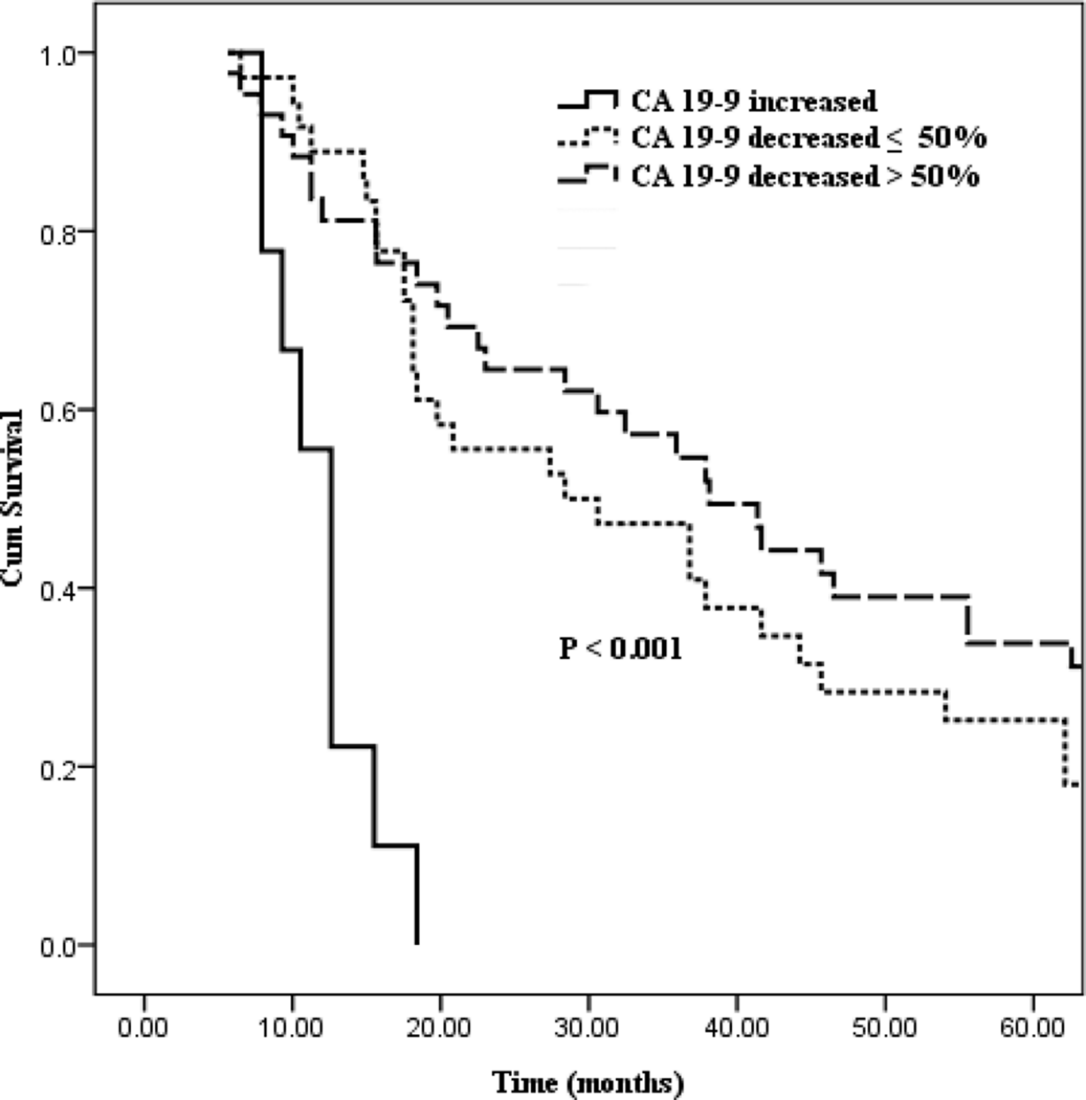
Kaplan–Meier curves comparing survival status stratified by preoperative to postoperative CA19-9 alterations (increased vs. decreased ≤ 50% vs. decreased > 50%; *P* < 0.001)

In order to examine the preoperative to postoperative CA19-9 changes on survival, we excluded those with preoperative CA19-9 levels within the base line point (≤ 37 U/ml, *n* = 10), and then we divided patients into 3 groups based on the transformation of CA19-9 levels: patients with postoperative CA19-9 increased (*n* = 9), patients with postoperative CA19-9 decreased ≤ 50% (*n* = 36), and patients with postoperative CA19-9 decreased > 50% (*n* = 43). Patients with increased postoperative CA19-9 levels had a poor median survival time of 12.6 months, while decreased postoperative CA19-9 levels ≤ 50% conferred to a median survival time of 28.4 months. Finally, in the last group with CA19-9 decreased > 50%, a relatively better median survival time of 38.1 months was expected (Figure 3, *P* < 0.001).

### Preoperative and postoperative CA19-9 levels with various tumor factors

Because the preoperative and postoperative CA19-9 cut-off points of 150 U/ml indicated the strongest predictive significance associated with survival. Then we further analyzed the relationships of preoperative and postoperative CA19-9 levels with other prognostic factors using the cut-off point of 150 U/ml (Table 3). In the univariate analysis, preoperative aspartate transaminase (AST) level > 40 U/L (*P* = 0.017), lymph node metastasis (*P* = 0.001), vascular invasion (*P* = 0.017), and early recurrence (*P* < 0.001) were associated with preoperative CA19-9 levels. In contrast, postoperative CA19-9 levels were obviously higher in those female patients (*P* = 0.002) with preoperative biliary drainage (*P* = 0.021), lymph node metastasis (*P* = 0.013), and early recurrence (*P* = 0.003).

**Table 3:** Univariate analysis of preoperative and postoperative CA19-9 levels with various tumor factors

	Preoperative CA19-9 levels*			Postoperative CA19-9 levels*		
Variables	≤ 150 (*n* = 57)	> 150 (*n* = 41)	*P* value	≤ 150 (*n* = 82)	> 150 (*n* = 16)	*P* value
Gender						
Male	25 (43.9)	26 (63.4)	0.056	45 (54.9)	2 (12.5)	0.002
Female	32 (56.1)	15 (36.6)		37 (45.1)	14 (87.5)	
Preoperative AST level > 40 U/L						
No	17 (29.8)	4 (9.8)	0.017	20 (24.4)	1 (6.3)	NS
Yes	40 (70.2)	37 (90.2)		62 (75.6)	15 (93.8)	
Preoperative biliary drainage						
No	51 (89.5)	32 (78)	NS	73 (89)	10 (62.5)	0.021
Yes	6 (10.5)	9 (22)		9 (11)	6 (37.5)	
Lymph node metastasis						
No	42 (73.7)	16 (39)	0.001	53 (64.6)	5 (31.2)	0.013
Yes	15 (26.3)	25 (61)		29 (35.4)	11 (68.8)	
Vascular invasion						
No	52 (91.2)	30 (73.2)	0.017	70 (85.4)	12 (75)	NS
Yes	5 (8.8)	11 (26.8)		12 (14.6)	4 (25)	
Early recurrence†						
No	51 (89.5)	19 (46.3)	< 0.001	64 (78)	6 (37.5)	0.003
Yes	6 (10.5)	22 (53.7)		18 (22)	10 (62.5)	

^*^ Present as number (%); ^†^Early recurrence defined as recurrence < 12 months after curative surgery;

Factors did not associated with CA19-9 levels included age, total hospital stay, preoperative CEA levels, preoperative CA125 levels, Bismuth-Corlette classification, caudate lobectomy, preoperative ALT levels, preoperative albumin levels, preoperative biliary drainage, portal vein embolization, tumor size, tumor differentiation, AJCC 7th T stage, surgical procedures, postoperative complications et al.

CA19-9: carbohydrate antigen 19-9; CA125: carbohydrate antigen 125; NS: not significant; AST: aspartate transaminase.

Then, a multivariate logistic regression model was conducted to analyze the significant factors (Table 4). The analysis identified patients with preoperative CA19-9 level > 150 U/ml were significantly associated with lymph node metastasis (OR [odds ratio] = 3.471, 95% CI 1.216–9.905; *P* = 0.020) and early recurrence (OR = 8.280, 95% CI 2.391–28.674; *P* = 0.001). Patients with preoperative CA19-9 > 150 U/ml are easy to suffer vascular invasion (OR = 3.643, 95% CI 0.955–13.897; *P* = 0.058), however, it did not reach statistical significance. Meanwhile, postoperative CA19-9 level > 150 U/ml was also correlated with early recurrence (OR = 4.006, 95% CI 1.107–14.459; *P* = 0.034). Similarly, patients have a higher tendency of lymph node metastasis (OR = 3.427, 95% CI 0.913–12.864; *P* = 0.068) when postoperative CA19-9 level > 150 U/ml, but it did not reach statistical significance.

**Table 4:** Multivariate analysis of significant factors among previous univariate analysis with CA19-9 levels

	Preoperative CA19-9 levels			Postoperative CA 19-9 levels		
Variables	odds ratio	95% CI	*P* value	odds ratio	95% CI	*P* value
Lymph node metastasis	3.471	1.216–9.905	0.020	3.427	0.913–12.864	0.068
Vascular invasion	3.643	0.955–13.897	0.058	-	-	-
Early recurrence	8.280	2.391–28.674	0.001	4.006	1.107–14.459	0.034

CA19-9: carbohydrate antigen 19-9.

## DISCUSSION

Currently, there are almost no studies in the literature that have comprehensively analyzed the prognostic influences of different cut-off points of preoperative and postoperative serum CA19-9 levels on survival of HCCH. Meanwhile, the ideal cut-off point in predicting survival and other prognostic factors, including early recurrence, has remained controversial. Furthermore, the predictive value of preoperative to postoperative CA19-9 alterations on survival has also not been well defined. Thus, it is now, our time to systematically elucidate the predictive values of preoperative and postoperative CA19-9 levels on survival and other prognostic factors, and which cut-off value of CA19-9 levels showed the strongest predictive value in HCCA.

To achieve the above goal, our current study divided the preoperative and postoperative CA19-9 levels into different groups based on the cut-off points of 37, 100, 150, 200, 400, 1000 U/ml, which were selected in the light of many previous studies and the distribution of CA19-9 levels in our current study. Our results revealed that increased preoperative CA19-9 levels were associated with poor survival outcomes. Of the six cut-off points, 150 U/ml revealed the strongest predictive value, which was consistent with the previous studies [16]. Different from previous studies, our study was not affected by elevated preoperative bilirubin levels because it was reported hyperbilirubinemia leads to a significant increase in CA19-9 level. Also different from former researches, we excluded those with CA19-9 nonsecretory, if were included in the research, the results would undoubtedly to be affected. Besides, our study also analyzed the relationships of different postoperative CA19-9 levels on survival and various tumor factors after curative resection. Similarly, the six cut-off points of postoperative CA19-9 levels were also identified as prognostic factors for HCCA, and the cut-off point of 150 U/ml simultaneously showed the strongest predictive value. To our knowledge, postoperative CA19-9 levels on survival had not been analyzed in previous literature. The postoperative CA19-9 levels of the 98 patients in our study were measured between 1 and 3 months after curative surgery. In viewing of this respect, conventional monitoring of the postoperative CA19-9 level within 1–3 months after surgery is a must and postoperative CA19-9 may be also recommended as a predictive factor to survival.

In addition, our study also assessed preoperative to postoperative CA19-9 alterations on survival. CA19-9 alterations could indirectly predict the disease evolution and increased postoperative CA19-9 is a reflection of tumor recurrence. Our results demonstrated that patients with increased postoperative CA19-9 tend to have poor survival outcome. Thus, attentions should also be paid on the preoperative to postoperative CA19-9 alterations so as to better predict the survival and tumor recurrence after surgery.

More importantly, our study also focused on the predictive values of preoperative and postoperative CA19-9 levels on various tumor factors before and after surgery. Our results showed that increased preoperative CA19-9 level has a higher tendency of lymph node metastasis, vascular invasion, and early recurrence. Meanwhile, early recurrence was also demonstrated as an independent factor with postoperative CA19-9 cut-off value of 150 U/ml. Many previous studies have reported patients with lymph node metastasis, vascular invasion and early recurrence are doubtlessly to have poor survival and have well been established as prognostic factors for survival after curative surgery of HCCA [22–26]. Thus, based on our results, we believe that CA19-9 levels can indirectly affect survival and increased preoperative and postoperative CA19-9 levels are associated with poor survival. Therefore, the preoperative CA19-9 levels should be taken into consideration when assessing the proper surgical procedures for relevant patients. Meanwhile, the postoperative CA19-9 levels should also be regularly monitored so as to help in early detection of tumor recurrence and lymph node metastasis.

Based on the retrospective nature of our current study with only 98 patients, future prospective analysis with more patient numbers or multi-center cooperation is undoubtedly needed to better clarify the prognostic effect of preoperative and postoperative CA19-9 levels on survival and various tumor factors, so as to better guide the treatment and predict survival.

In conclusion, patients with increased preoperative and postoperative CA19-9 levels have a higher tendency of lymph node metastasis and early recurrence in patients with resectable hilar cholangiocarcinoma. On the one hand, increased CA19-9 levels can directly predict poor survival outcome. On the other hand, CA19-9 levels can impact lymph node metastasis, vascular invasion, early recurrence, and then indirectly affect survival.

## MATERIALS AND METHODS

### Patient selection

From January 1995 to December 2014, a total of 98 patients diagnosed with HCCA who underwent curative surgery (including R0 and R1 resection) and with exploitable CA19-9 levels from West China Hospital of Sichuan University were retrospectively included. Patients with R2 resection and palliative surgery were excluded. Both patients with hyperbilirubinemia (preoperative total bilirubin levels > 34.2 umol/l after biliary drainage) and those with CA19-9 nonsecretory (preoperative serum CA19-9 levels < 5 U/ml) were also excluded. None of the included patients accepted any anti-cancer treatment prior to surgery. A diagnosis of hilar bile duct adenocarcinoma was acknowledged histologically in all cases.

### Data collection

Serum CA19-9 level was detected using Roche chemiluminescence immunoassay kit. Preoperative serum CA19-9 was detected just before the surgery so as to evade the effect of hyperbilirubinemia on CA19-9 level. Postoperative serum CA19-9 level was measured between 1 and 3 months after surgery. Preoperative biliary drainage was conducted on patients with obstructive jaundice using endoscopic retrograde biliary drainage or percutaneous transhepatic biliary drainage. Patients with preoperative total bilirubin levels > 34.2 umol/l after biliary drainage were excluded. Both preoperative and postoperative CA19-9 levels were divided as ≤ 37 U/ml vs. > 37 U/ml, ≤ 100 U/ml vs. > 100 U/ml, ≤ 150 U/ml vs. > 150 U/ml, ≤ 200 U/ml vs. > 200 U/ml, ≤ 400 U/ml vs. > 400 U/ml, ≤ 1000 U/ml vs. > 1000 U/ml. These cut-off values were selected in the light of many previous studies and the distribution of CA19-9 levels in our current study [15, 16, 22, 27, 28].

### Postoperative follow-up

Patients were strictly monitored at outpatient department after surgery. Regular laboratory tests such as liver functions, and tumor markers were conducted and abdominal ultrasound was routinely proceed every 2–3 months during the first year, then 3–6 months yearly thereafter. Computed tomography scan of abdomen or magnetic resonance imaging test was further adopted if patients were suspected as tumor recurrence.

### Statistical analysis

Patient characteristics were conveyed using frequency or descriptive analysis. The Kaplan-Meier analysis and log-rank test were adopted to analyze the difference in overall survival between groups divided based on the six cut-off points of preoperative and postoperative CA19-9 levels listed above. Preoperative to postoperative CA19-9 changes on survival were simultaneously analyzed using the same methods. The CA19-9 cut-off point showing the strongest significance was further examined using the univariate and multivariate logistic regression models, and many preoperative and postoperative factors including early recurrence were analyzed so as to identify the independent factors associated with CA19-9 levels. The *P value* < 0.05 was deemed as significant. The statistical analysis was outlined using the SPSS version16.0 (SPSS Inc. Chicago, IL, USA).

## Abbreviations

HCCA: Hilar cholangiocarcinoma
CA19-9: Carbohydrate antigen 19-9
